# Complete Coding Sequences of Eastern Equine Encephalitis Virus and Venezuelan Equine Encephalitis Virus Strains Isolated from Human Cases

**DOI:** 10.1128/genomeA.00243-15

**Published:** 2015-04-23

**Authors:** Guo-Yun Yu, Michael R. Wiley, Jeffrey R. Kugelman, Jason T. Ladner, Brett F. Beitzel, Lori T. Eccleston, Elaine M. Morazzani, Pamela J. Glass, Gustavo F. Palacios

**Affiliations:** a Center for Genome Sciences, United States Army Medical Research Institute of Infectious Diseases, Fort Detrick, Maryland, USA; b Virology Division, United States Army Medical Research Institute of Infectious Diseases, Fort Detrick, Maryland, USA

## Abstract

We obtained the complete coding genome of an eastern equine encephalitis virus (EEEV) strain, EEEV V105-00210, and the complete genome of a Venezuelan equine encephalitis virus (VEEV) strain, VEEV INH-9813. They were obtained from human cases and are proposed as reference challenge strains for vaccine and therapeutic development in animal models.

## GENOME ANNOUNCEMENT

Venezuelan equine encephalitis (VEEV) and eastern equine encephalitis (EEEV) viruses belong to the *Alphavirus* genus within the *Togaviridae* family. They are positive-strand RNA viruses causing fatal encephalitis in horses and humans. VEEV has mainly been found in South and Central America, causing extensive epizootic infections in horses and widespread epidemic infections in humans (1, 2). EEEV has primarily been found in eastern North America but has also been isolated in South America (3, 4). Although EEEV isolates from South America are avirulent to humans, North American isolates are highly virulent, being the most deadly mosquito-borne pathogen in North America (4), with a fatality rate estimated at 35 to 75% (5). Moreover, EEEV infection causes neurologic impairments in its survivors (6). Both viruses are able to infect many animals, including mammals, birds, reptiles, and amphibians. In nature, they cycle between animal hosts and mosquito vectors and are transmitted to humans through the bite of an infected mosquito (7–9). Over the past decade, EEEV has resurged in the northeastern United States and expanded northward into new regions, including northern New England and eastern Canada (6, 10). These viruses are considered significant threats to public health. Additionally, they can potentially be used as biological weapons, since they are highly infectious in aerosol form (11). No licensed therapeutics or vaccines for treating human infections are currently available (12). To facilitate vaccine and drug research efforts (12–15), we provide the viral genome sequences of the two challenge stocks derived from the two human isolates.

VEEV INH-9813 (IC strain) was isolated from a serum sample from a human patient in Venezuela during the 1995 outbreak (16). EEEV V105-00210 was isolated from the brain cortex of a fatal human case in MA in 2005 (17). The viruses were isolated by a single passage on Vero cells (V-1) (17). The V-1 materials were used to prepare a master stock (V-2), which was used to infect Vero cells for the preparation of a sucrose-purified virus stock (V-3). RNA was extracted from the sucrose-purified virus preparations using the TRIzol LS procedure (Invitrogen) and used for cDNA synthesis by sequence-independent single-primer amplification (SISPA) (18, 19). Purified cDNA was amplified with MyTaq DNA polymerase (Bioline, Taunton, MA), and the purified PCR products were fragmented. Libraries were prepared with the TruSeq DNA sample preparation kit (Illumina, San Diego, CA). After quantification by real-time PCR with the Kapa qPCR kit (Kapa Biosystems, Woburn, MA), the libraries were diluted to 10 nM and sequenced on an Illumina MiSeq with a 200-bp paired-end protocol.

For VEEV INH-9813, the complete genome sequence (20) shared 99.91% (10 mismatches, 4 amino acid [aa] changes) base identity with the genome of an ID serotype isolated from mosquitoes in Delta Amacuro, Venezuela, in 1973 (accession no. KC344512.1). The complete genome (20) sequence obtained for EEEV V105-00210 was nearly identical (99.98%; 2 mismatches, 0 aa changes) to the sequence of a strain isolated from mosquitoes in Florida in 2005 (accession no. KJ469556.1).

### Nucleotide sequence accession numbers. 

The GenBank accession numbers for INH-9813 and V105-00210 are KP282671 and KP282670, respectively.
